# The Simultaneous Application of Transcranial Magnetic Stimulation and Virtual Reality to Treat Cognitive Deficits Among Stroke Patients: A Randomized Controlled Trial

**DOI:** 10.7759/cureus.62434

**Published:** 2024-06-15

**Authors:** Priya Chauhan, Sanjib K Das, SD Shahanawaz

**Affiliations:** 1 Department of Physiotherapy, Amity Institute of Health Allied Sciences, Noida, IND; 2 Department of Physiotherapy, College of Applied Medical Sciences, University of Hail, Hail, SAU

**Keywords:** neuroplasticity, cognitive rehabilitation, virtual reality training, repetitive transcranial magnetic stimulation, cognitive deficits, stroke

## Abstract

Background and objective

Integrating virtual reality (VR) and transcranial magnetic stimulation (TMS) offers a promising strategy for stroke rehabilitation, as it specifically focuses on reorganizing neural connections and activating brain activity in the cortex. The main goal is to create equitable connections between the brain's two hemispheres to enhance the execution of voluntary movements by stimulating the central executive network (CEN) to strengthen both motor and cognitive abilities. This study aims to propose a therapeutic approach that can improve cognitive recovery and overall quality of life in patients after a stroke.

Methods

A total of 69 participants were enrolled in the study based on certain inclusion and exclusion criteria. The patients underwent pre-assessment and were randomly allocated into three groups: Group 1 received simultaneous repetitive TMS (rTMS) and virtual reality treatment (VRT), Group 2 received rTMS combined with sham VRT, and Group 3 received sham stimulation with VRT, in a 1:1:1 ratio using opaque, sealed, and stapled envelopes (SNOSE). Post-assessment was carried out using the same measures: the National Institutes of Health Stroke Scale (NIHSS), Addenbrooke's Cognitive Test (ACE III), and Montreal Cognitive Assessment (MOCA). Statistical analysis was conducted to determine the specific outcomes. Data analysis was carried out using IBM SPSS Statistics version 29 (IBM Corp., Armonk, NY), employing student's t-test for within-group comparisons and repeated measures ANOVA for between-group comparisons. The significance level was set at 5%.

Results

The results demonstrated statistical significance in NIHSS scores across all treatment groups (p<0.001). Regarding cognitive outcomes, improvements were observed in memory, language, and overall cognitive performance (ACE III) within all groups (p<0.05), with significant between-group outcomes (p = 0.009, p = 0.01, p = 0.004, respectively), suggesting variations in treatment effects across cognitive domains. However, no significant differences between groups were found in terms of fluency and visuospatial skills (p = 0.49, p = 0.13), indicating no treatment effects in these domains.

Conclusions

Based on our findings, the combined intervention involving rTMS and VRT, compared to sham treatments, demonstrates promising outcomes in alleviating stroke severity and improving specific cognitive functions such as memory, language, and overall cognitive performance. Additionally, the combined administration offers a more effective therapy than when they are administered separately.

## Introduction

Stroke accounts for a significant chronic debility burden on a global scale, impacting a considerable number of people annually. Globally, there were 12.2 million stroke cases, 101 million prevalent cases, 143 million disability-adjusted life years (DALYs) due to stroke, and 6.55 million deaths in 2019. Stroke was the second-leading cause of death worldwide, accounting for 11.6% of total deaths. While age-standardized rates decreased during 1990-2019, the absolute numbers increased significantly. In India, stroke incidence ranges from 108 to 172 per 100,000, with a crude prevalence of 26-757 per 100,000 [1]. Despite the remarkable progress made in the domain of stroke rehabilitation, a considerable proportion of individuals continue to experience severe sensorimotor and cognitive impairments, leading to a profound effect on their overall quality of life and ability to perform daily activities autonomously.

Throughout history, a significant shift has occurred in the approach to restoring stroke patients to health, characterized by a departure from traditional therapies and the adoption of more targeted and effective methodologies. This paradigm shift is attributed to the awareness that conventional remedies have demonstrated limited efficacy in facilitating complete functional recovery, particularly in cases of significant impairment [2]. Stroke patients who suffer from cognitive impairments in addition to motor impairments face significant challenges as they become dependent on others and are incapable of independently carrying out their daily tasks. Cognitive deficits following a stroke may manifest as difficulties with executive function, attention, language, memory, abstract thinking, time and place orientation, visuospatial skills, and computing ability. Even months after the initial stroke, a significant number of stroke survivors continue to experience functional limitations, which further impact their quality of life and daily activities [3].

Various rehabilitation interventions have been implemented to promote and facilitate the restoration of cognitive functions in this population. Recent rehabilitation techniques focus on a top-to-bottom approach by targeting the impacted area. Transcranial magnetic stimulation (TMS) is a noninvasive procedure that uses the application of magnetic fields to stimulate nerve cells in the brain to improve various symptoms. It involves delivering repetitive magnetic pulses and is hence termed repetitive TMS or rTMS. Its mechanism of action relies on stimulating the functional neural networks over the stimulation site [4]. Virtual reality (VR) also creates a virtual environment that leads to integrated signals between motor intention, execution, and multi-sensory feedback, leading to a sense of control of one’s movements. This also results in the activation of the primary motor cortex (M1) and secondary motor areas, including the premotor cortex, supplementary motor area, and parietal cortices [5].

Of late, VR training and rTMS have emerged as potentially effective therapeutic modalities [6-7]. Studies have shown that the simultaneous application of distinct therapeutic methods can produce a synergistic outcome, ultimately enhancing prognoses for stroke patients. As both therapeutic techniques activate the central executive network (CEN), we hypothesized that rTMS along with simultaneous VR treatment (VRT) might specifically improve cognitive control in the stroke population [8]. Thus, combining these two therapeutic approaches might stimulate CEN and have complementary effects on neural remodeling and cortical excitability, individually. Also, the incorporation of multi-sensory experiences might lead to more extensive and enduring effects on cognitive control and sensorimotor function in stroke patients. It has been speculated that combining VRT and rTMS can effectively stimulate the damaged hemisphere while suppressing the unaffected hemisphere, and thus the fractured inter-hemispheric rivalry could be targeted. It is critical to establish fair inter-hemispheric connections to facilitate the effective implementation of various cognitive parameters. Hence, this study aimed to analyze the combined effect of the simultaneous application of rTMS and VRT.

## Materials and methods

Study design

**Figure 1:**
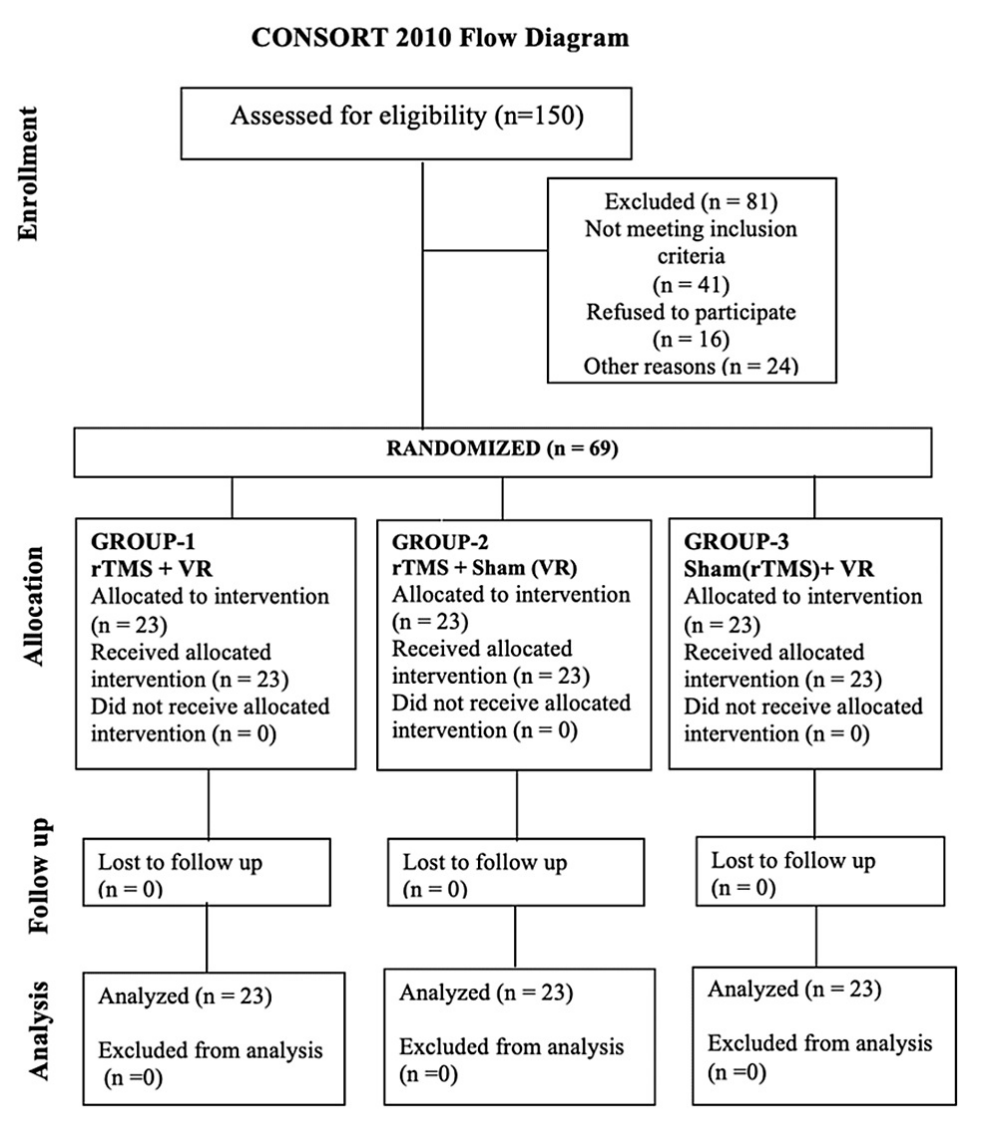
CONSORT flow diagram CONSORT: Consolidated Standards of Reporting Trials; rTMS: repetitive transcranial magnetic stimulation; VR: virtual reality

We conducted a three-week, interventional, prospective, randomized controlled, parallel clinical trial designed per the CONSORT (Consolidated Standards of Reporting Trials) 2010 guidelines (Figure 1). It assessed the effectiveness of TMS and VRT with a placebo in treating sensorimotor and cognitive deficits in stroke patients. The study was carried out at a tertiary hospital in Noida, from July 2023 to April 2024, spanning one year.

Sample size

The sample size was calculated using G Power version 3.1.2 software. FMA-UE was used as the data analysis parameter, and a two-sided test with α = 0.05 and accuracy level (test 1-β) = 0.8 was used. N = 60 was the approximate total sample size. With 23 individuals in each group, the total sample size was N = 69, adjusting for an anticipated 12% dropout rate.

Ethical considerations

All participants signed a written consent and were informed about the study in detail. The study protocol was based on Standard Planning Recommendations for Interventional Research (SPIRIT) principles and was registered with the Indian Clinical Trials Registry (CTRI/07/055713, 2023). The ethical committee of Amity University, Noida, approved the study (approval no: AUUP/IEC/JULY/2022).

Study protocol and participant selection criteria

Participants of all genders aged 40-70 years who were willing to participate in the study and fulfilled the inclusion criteria of unilateral hemispheric stroke with a duration of two to nine months were recruited. On the paretic side, participants had to exhibit motor evoked potentials (MEPs), at least 10 degrees of active finger movement, and 20 degrees of active wrist extension. This enabled the participation of all individuals in virtual reality training without any limitations. Cognitive function was assessed using the Montreal Cognitive Assessment (MOCA) Scale, with a minimum score of 18 points and a cutoff score below 15 points on the National Institutes of Health Stroke Scale (NIHSS). The exclusion criteria were as follows: any history of neurological or psychiatric disease within two years that may affect the study, paralysis on both sides, severe aphasia, taking drugs that could change the excitability of the cerebral cortex, and any contraindications to TMS and/or MRI, such as implanted cardiac pacemaker, metallic foreign bodies, any cranial metallic implants, history of neurosurgery, and other electronic devices implanted in the body that are contraindicated for TMS treatment.

A total of 69 participants who met all the inclusion and exclusion criteria and provided informed consent were enrolled in the study. The physician reviewed each subject's medical history and scores and determined their eligibility to participate. They were randomly assigned to either of the following three groups - Group 1: rTMS along with virtual reality training and conventional therapy; Group 2: rTMS with sham virtual reality training and conventional therapy; and Group 3: sham rTMS with virtual reality training and conventional therapy. The patients were pre-assessed and post-assessed for the following outcome measures: NIHSS, MOCA, and Addenbrooke's Cognitive Assessment III (ACE III). After the intervention period, appropriate statistical parameters were analyzed, and results were obtained. All the participants would undergo 12 sessions over three weeks (Monday, Tuesday, Wednesday, and Friday); the rest of the days were set aside for conventional therapy daily for three weeks.

Randomization

Randomization was used in conjunction with the selection process to recruit patients. Patients were recruited according to the selection criteria by using the block randomization method. They would be allocated into either of the three groups in a 1:1:1 ratio using sequentially, numbered, opaque, sealed, and stapled envelopes (SNOSE).

Procedure

Group 1: Simultaneous rTMS and VRT

The participants were scheduled to receive 25 minutes of rTMS while actively engaging in three VRT games. The precise location of the target was determined on the first day. rTMS stimulation involved the use of a magnetic stimulator attached to a figure-of-eight coil. The intensity level for all participants was set at 110% of their resting motor threshold (RMT). The threshold was determined by applying tangential stimulation to the convex part of the skull by using a figure-of-eight coil. This was done to evaluate the response of the first dorsal interosseous muscle, specifically in the DLPFC. During each daily session, rTMS was administered at a frequency of 10 Hz for five seconds, followed by a stimulation-free interval of 25 seconds. This resulted in a total of 2,000 pulses delivered in 12 separate sessions throughout the day. The intensity was augmented as necessary. Each participant received a total of 12 sessions over three weeks on Mondays, Tuesdays, Wednesdays, and Fridays. Additionally, physical therapy was provided on all of these days for the whole three-week duration.

Simultaneously, for individuals who were engaged in virtual gaming, a VRT headset was put over the rTMS unit. The gaming setup comprised a computer and sound system, accompanied by a Cy-Wee Z game controller showcased on a 32-inch monitor. The controller was equipped with gyroscopes, magnetic sensors, and accelerometers, enabling users to explore three-dimensional space while accurately detecting depth. The game controller was outfitted with a specialized handle that facilitated effortless movement on either side of the body. Throughout the treatment course, patients were engaged in VRT activities, including Music Capture, Bounce, Mosquito Killer, Bejeweled, Balloon Shooting, Ten Pin Bowling, Solitaire, Air Hockey, and Mahjong. The level of difficulty was contingent upon the players. This setup was specifically designed to promote the active involvement of participants during virtual sessions. The duration of treatment was fixed for eight minutes each, with a two-minute interval between each game comprising a total of 30 minutes.

Group 2: rTMS Combined With Sham VRT

rTMS was applied using a previously elucidated procedure, applicable for administering stimulation over the specified area. For the sham VR session, a similar strategy used for real VR was used. The person sat comfortably on the chair and the VR session was constantly monitored by the therapist. Per the VR-CORE clinical trial guidelines, the sham VR session consisted of a vigorous and active placebo of a non-immersive, two-dimensional (2D) visual game displayed within a commercial VR headgear. Participants were provided with an all-in-one head-mounted VR device that was user-friendly and had minimal visual latency. The hardware was capable of displaying 2D games (sham VR); however, the VR games were chosen in a manner that ensured the participants' movements did not exceed the midline and were played randomly. Therefore, the majority of the hemiplegic upper limb's actions during sham treatment consisted of striking the selection button and reaching for the buttons. The duration of treatment was fixed for eight minutes each, with a two-minute interval between each game comprising a total of 30 minutes while receiving the rTMS therapy.

Group3: Sham Stimulation With VRT

The participants in this group were connected to an rTMS component with figure-eight dummy coils as part of the sham stimulation. The sham coil is ineffective in extracting power and hence fails to deliver any stimulation to the target area. Instead, it emits a feeble force that spreads throughout a wide area surrounding the target, impeding the activation of the motor cortex. Because the dummy coil could not draw power, it could not stimulate the target; instead, it released a weak force that prevented activation of the motor cortex in a large area around the target. Participants were simultaneously given a 30-minute VRT under the supervision of a therapist using a previously elucidated procedure.

Conventional Therapy

The conventional therapy protocol for stroke rehabilitation was designed according to the FITT principle and consisted of 30-minute sessions five times a week for three weeks. It begins with a five-minute warm-up followed by 10 minutes of strength training for the upper extremity and core and 10 minutes of strength training for the lower extremity. It ends with a minute cool-down that involves gentle stretching of the arms, legs, and back, holding each stretch for 15-30 seconds to focus on muscle relaxation.

Outcome measures

National Institutes of Health Stroke Scale (NIHSS)

The NIHSS is a measure of somatosensory function in individuals who have experienced a stroke. It comprises 11 items; two of the 11 items are physician-administered assessments of the patient’s upper and lower extremity range of motion. The diagnosis for the other nine items is determined by the physician (e.g., gaze, facial paralysis, dysarthria, level of consciousness). All items are rated on a 3-point scale (0= normal, 2 = severe impairment). This measure has been demonstrated to have good reliability and validity.

Addenbrooke's Cognitive Examination III (ACE III)

ACE III is a comprehensive screening examination that assesses an individual's cognitive abilities in multiple domains. The test evaluates attention (18 points), memory (26 points), fluency (14 points), language (26 points), and visuospatial skills (16 points), with a maximum possible score of 100 points. This assessment instrument is beneficial for the identification of cognitive impairment and can expedite the diagnostic process following a stroke. Additionally, it can inform the implementation of cognitive rehabilitation strategies that are customized to the individual's performance and requirements.

Statistical analysis

Data analysis and interpretation were conducted using IBM SPSS Statistics version 29 (IBM Corp., Armonk, NY). Descriptive statistics, such as percentage, mean, and standard deviation (SD), were computed for the relevant variables. After verifying the data's normal distribution using the Shapiro-Wilk normality test, we proceeded to analyze it using the paired t-test for within-group comparisons and repeated measures ANOVA for between-group comparisons. The significance level was established at 5%. Any p-values below 0.05 were considered statistically significant.

## Results

Table 1 provides the demographic data of all three groups as well as an overview of all the clinical characteristics of participants, categorized by gender and treatment group. Gender distribution across the groups showed a predominance of male participants, with proportions ranging from 78.2% to 82.6%. Mean age, time since stroke, height, and weight are presented with standard deviations, reflecting the variability within each group.

**Table 1:** Demographic characteristics of the participants MOCA: Montreal Cognitive Assessment; SD: standard deviation

Variables	Group 1	Group 2	Group 3
Gender, n (%)			
Female	4 (17.4%)	5 (21.7%)	5 (21.7%)
Male	19 (82.6%)	18 (78.2%)	18 (78.2%)
Age, years, mean ± SD	53.52 ± 10.38	53.70 ± 9.48	53.43 ± 9.93
Time since the stroke, mean ± SD	4.57 ± 1.24	3.96 ± 1.11	4.83 ± 0.94
Height, CM, mean ± SD	167.65 ± 9.10	167.26 ± 9.24	169.83 ± 8.66
Weight, KG, mean ± SD	67.91 ± 9.22	67.96 ± 10.53	70.39 ± 9.40
MOCA scale score, mean ± SD	19.71 ± 4.52	20.57 ± 2.04	20.52 ± 2.04

The outcome measures for the three groups were assessed using NIHSS, measuring pre- and post-treatment scores. In Group 1, the mean NIHSS score significantly decreased from 16.13 (SD = 15.62) before treatment to 14.91 (SD = 1.13) after treatment, with a significant p-value of <0.001 within the group based on the student’s t-test. Group 2 showed a decrease in mean NIHSS score from 9.35 (SD = 1.64) to 6.74 (SD = 1.79), also with a significant p-value of <0.001. Similarly, Group 3 exhibited a decrease from 9.57 (SD = 1.38) to 7.39 (SD = 1.50), again with a significant p-value of <0.001. Furthermore, an ANOVA test between groups demonstrated a significant F-value of 3.94 (p = 0.024), indicating differences in outcomes among the treatment groups. These findings suggest that all three treatment approaches led to significant improvements in NIHSS scores, with some variations observed between the groups. Tables 2 show the group analysis for stroke severity based on NIHSS.

**Table 2:** Within-group and between-group analysis of the severity of stroke *P<0.05 ANOVA: analysis of variance; NIHSS: National Institutes of Health Stroke Scale; SD: standard deviation

Outcome measure	Group	Variable	Mean	SD	Student's t-test (within-group)	F-value	ANOVA (between-group)
NIHSS	A	Pre	16.13	15.62	<0.001*	3.94	0.024*
		Post	14.91	1.13			
	B	Pre	9.35	1.64	<0.001*		
		Post	6.74	1.79			
	C	Pre	9.57	1.38	<0.001*		
		Post	7.39	1.5			

The outcomes across various cognitive domains were assessed using different measures including attention, memory, fluency, language, visuospatial skills, and the ACE III. Table 3 shows the group analysis for various cognitive domains. For attention, Group 1 had a mean score of 16.13 (SD = 15.62), which decreased slightly to 14.91 (SD = 1.13) post-treatment, although this change was not statistically significant (p = 0.715). Group 2 demonstrated a notable increase in mean attention scores from 12.83 (SD = 1.78) before treatment to 14.74 (SD = 0.92) post-treatment (p<0.001). Similarly, Group 3 showed a significant improvement in attention, with mean scores rising from 13.09 (SD = 1.74) pre-treatment to 14.35 (SD = 1.19) post-treatment (p<0.001). Additionally, ANOVA analysis revealed significant between-group differences F-value of 1.64 (p = 0.20), indicating non-significant variations in the effectiveness of the different treatment approaches on attention levels.

**Table 3:** Within-group and between-group analysis of ACE III scores and sub-scores *P<0.05 ACE III: The Addenbrooke's Cognitive Examination III; ANOVA: analysis of variance; SD: standard deviation

Outcome measure	Group	Variable	Mean	SD	Student's t-test (two-sided significance)	ANOVA F-value (between-group)	P-value
Attention	A	Pre	16.13	15.62	0.715	1.64	0.20
		Post	14.91	1.13			
	B	Pre	12.83	1.78	<0.001*		
		Post	14.74	0.92			
	C	Pre	13.09	1.74	<0.001*		
		Post	14.35	1.19			
Memory	A	Pre	20.17	2.57	<0.001*	5.09	0.009*
		Post	22.65	1.75			
	B	Pre	19.22	2.47	<0.001*		
		Post	21.65	0.71			
	C	Pre	18.35	2.10	<0.001*		
		Post	21.61	1.08			
Fluency	A	Pre	10.30	1.36	<0.001*	0.70	0.49
		Post	12.61	0.78			
	B	Pre	10.09	1.35	<0.001*		
		Post	12.39	0.50			
	C	Pre	10.48	1.38	<0.001*		
		Post	12.43	0.66			
Language	A	Pre	20.52	2.98	<0.001*	4.34	0.01*
		Post	22.57	1.44			
	B	Pre	20.78	2.73	0.079		
		Post	21.87	1.22			
	C	Pre	19.96	2.67	0.005*		
		Post	21.57	0.79			
Visuospatial	A	Pre	12.04	2.16	<0.001*	2.05	0.13
		Post	14.48	0.73			
	B	Pre	11.52	1.78	<0.001*		
		Post	14.13	0.34			
	C	Pre	11.65	1.72	<0.001*		
		Post	14.26	0.62			
Addenbrooke's Cognitive Test total	A	Pre	75.84	7.69	<0.001*	5.93	0.004*
		Post	87.46	4.01			
	B	Pre	74.40	6.26	<0.001*		
		Post	84.99	1.82			
	C	Pre	73.39	6.11	<0.001*		
		Post	84.76	2.60			

In memory, language, and ACE III, significant improvements were observed within all three groups from pre- to post-treatment with significant values of less than p = 0.05. Additionally, the ANOVA tests indicated significant differences between the groups for memory, language, and ACE III (p = 0.009, 0.01, and 0.004 respectively), suggesting variations in treatment effects across cognitive domains. Additionally, the ANOVA tests indicated non-significant differences between groups in fluency (p = 0.49) and visuospatial parameters (p = 0.13), suggesting no variations in treatment effects across these domains. Overall, the findings suggest that the treatments had significant positive effects on various cognitive functions, with some differences noted between the treatment groups.

## Discussion

The most important finding of our research is that the combined use of TMS and VRT exhibits a more significant improvement in cognitive parameters compared to each intervention used separately in patients with subacute stroke. All three groups showed improvements in NIHSS and ACE scores during the study. However, variations were observed among the groups in terms of the specific sub-scores [9]. Physiological changes observed following a stroke indicate that increased excitability in the opposite hemisphere of the brain and a decrease in gamma-aminobutyric acid type A receptors may start within a week after the damage and last for up to eight months [10]. Research has also demonstrated that the heightened activation of the variant is particularly significant during the initial phase after a stroke. The mechanisms driving dynamic neuroplasticity may not always be advantageous for the process of healing and can potentially lead to adverse repercussions with recovery. We specifically chose patients who experienced a subacute stroke within that specific time frame. Our findings demonstrate a notable enhancement in cognitive indices following the combined use of VRT and rTMS, in comparison to the other groups that received sham therapy.

Our findings align with the research results reported by Pinter et al. who concluded that applying high-frequency rTMS on the same side of the brain as the injury, namely on DLPFC, can have an immediate positive effect on post-stroke cognitive impairment. Their study also indicated that the simultaneous application of rTMS might significantly enhance upper limb function, ADL, and overall quality of life in individuals with subacute stroke [11]. This combination approach may offer an effective rehabilitation treatment for subacute stroke patients. The observed cognitive gain following rTMS and VR may be attributed to the stimulation's quick and direct impact on the targeted region. This effect can affect interneurons, resulting in an increased flow of blood and high neurotransmitter levels such as dopamine, acetylcholine, serotonin, and norepinephrine [12].

The physiological basis for these improvements may be explained by the influence of synaptic transmission by rTMS resulting in altering the effectiveness of synaptic communication, specifically by strengthening synaptic connections, a phenomenon referred to as long-term potentiation [13]. The observed facilitatory aftereffects of cortical excitability following rTMS and VR are associated with an increase in γ-aminobutyric acid-mediated inhibition and an enhancement of N-methyl-d-aspartate receptor activity, particularly when multiple rTMS sessions are administered. This indicates that following rTMS and VR sessions, there are noticeable alterations in cortical excitability, which refers to the capacity of neurons in the cerebral cortex to respond to stimuli. These alterations are thought to be simple as they enable a greater number of neurons to function [14]. Stagg et al. also described a positive relationship between physiological measures and levels of GABA and glutamate in the human cortex [15].

The current study also demonstrates that rTMS therapy along with VRT has improved memory and language. This can be explained by rTMS's potential to increase memory and learning by modifying synapse strength. The reason for this is that synaptic neural activity is essential in controlling memory and learning processes [16]. DLPFC is a brain region that shows a connection between a decrease in GABA levels and the decline in cognitive function associated with stroke [17]. Li et al. have shown in their review that using traditional high-frequency rTMS to target the DLPFC can improve overall cognitive function, which includes memory and language [18]. DLPFC is a brain region that exhibits a correlation between GABA levels and a reduction in cognitive recall. GABA functions as the principal inhibitory neurotransmitter. The lack of GABA in the DLPFC disrupts the balance between neuronal excitation and inhibition, resulting in increased neural activity, potential harm to the brain, and a loss of cognitive function [19,20]. The interconnection between different regions and networks in the brain may help explain the neuronal pathways that are activated during rTMS stimulation.

A recent study conducted by Kim et al. has shown a significant association between the hippocampus and the fronto-parietal network (FPN) of the brain regarding memory retrieval [21]. The reason for this is that the DLPFC plays a crucial role in the functioning of the FPN. By applying magnetic stimulation to the DLPFC, it is possible to improve the connectivity within the FPN, resulting in stronger connections between the FPN and the hippocampus, consequently enhancing memory [22]. This particular mechanism may have relevance in other domains of memory, and further investigation exploring this process across several subtypes of memory could help elucidate the precision and suitability of this prospective approach. Further studies on a larger population that involve comparing the stimulation effect on various target sites can be conducted to evaluate the effectiveness of the targeted area and its physiological connection in improving various other cognitive and sensory-motor parameters as well as correlating the impact of various physical activities and immersive technologies for the same.

Limitations**

The study's limitations pertain to variations in the proportions of male and female participants across all three categories. Furthermore, it is possible to expand the sample size of this research and incorporate more precise quantitative outcome measures to substantiate the physiological improvement across various groups. Additionally, this study had shortcomings in terms of follow-up measures; therefore, additional research may be warranted to examine the durability of implemented improvements.

## Conclusions

According to the findings of this study, combined rTMS and VRT for cognitive impairments in stroke patients is highly efficient and has produced encouraging outcomes. The combination therapy has shown substantial enhancements in cognitive skills, such as attention, memory, and executive functions, in comparison to the sham treatment group. Patients who received the combined rTMS and VRT demonstrated accelerated cognitive recovery over a fixed period. This combination utilizes the neuroplasticity-enhancing benefits of rTMS and VRT, thereby providing a new and successful rehabilitation strategy. These results indicate that the combined approach has the potential to significantly enhance conventional stroke rehabilitation methods.
